# Empirical validation of an agent-based model of wood markets in Switzerland

**DOI:** 10.1371/journal.pone.0190605

**Published:** 2018-01-19

**Authors:** Stefan Holm, Lorenz M. Hilty, Renato Lemm, Oliver Thees

**Affiliations:** 1 Swiss Federal Institute for Forest, Snow and Landscape Research WSL, Birmensdorf, Switzerland; 2 Department of Informatics, University of Zurich, Zürich, Switzerland; 3 Swiss Federal Laboratories for Materials Science and Technology EMPA, St. Gallen, Switzerland; University of Vermont, UNITED STATES

## Abstract

We present an agent-based model of wood markets and show our efforts to validate this model using empirical data from different sources, including interviews, workshops, experiments, and official statistics. Own surveys closed gaps where data was not available. Our approach to model validation used a variety of techniques, including the replication of historical production amounts, prices, and survey results, as well as a historical case study of a large sawmill entering the market and becoming insolvent only a few years later. Validating the model using this case provided additional insights, showing how the model can be used to simulate scenarios of resource availability and resource allocation. We conclude that the outcome of the rigorous validation qualifies the model to simulate scenarios concerning resource availability and allocation in our study region.

## 1 Introduction

Agent-based Modeling (ABM) is a bottom-up modeling approach, where "*a system is modeled as a collection of autonomous decision-making entities called agents*" [1]. This requires that the system under study can be decomposed into its constituent units. ABM is especially beneficial if such decomposition and the description of the resulting units leads to a natural representation of the system [1,2]. Important advantages of using ABM are the possibilities of modeling each agent individually and capturing emergent behavior at any level of aggregation [1,2].

While the reasons for modeling and simulation are manifold [3], Kelly et al. [4] identified two model purposes for which ABM is the most appropriate approach, namely system understanding and social learning. While prediction is often assumed to be the main purpose of modeling and simulation [3], this is in fact seldom the case for agent-based models: Heath et al. [5] analyzed studies that used ABM and were published between 1998 and 2008, and did not find a single study that uses an agent-based model for prediction as the main purpose. However, there are different notions of the term "prediction". Heath et al. [5] state that if a model is used as a predictor, "it is used like a calculator to provide clear and concise predictions about the system", in contrast to its use as a mediator, when there is less understanding about the real system and "the simulation provides insight into the system, but is not a complete representation of how that system actually behaves". Kelly et al. [4] differentiate between prediction and forecast, where prediction leads to "if-then" results (exogenous factors of the model are known or assumed), and forecast, where statements regarding the future are made without knowledge of the exogenous factors of the system (everything is calculated inside the model). The differences between prediction and forecast are field-specific, as can be seen in the example of seismology, where "A prediction is a definitive and specific statement about when and where an earthquake will strike […] Whereas a forecast is a probabilistic statement, usually over a longer timescale" [6]. In this article, we use the term "prediction" according to the definition of Kelly et al. [4], whereas, according to Heath et al. [5], our model would be a "mediator".

ABM has been used in a multitude of disciplines, such as social sciences, economics, biology, traffic simulation, and crime analysis [2,5,7]. While early agent-based models were rather theoretical and abstract [8], e.g., Schelling’s segregation model [9], or Axelrod’s modeling of different strategies in the Prisoner’s Dilemma [10], large and complex systems are modeled and simulated today to draw conclusions for, e.g., policy making [7,11]. This makes model validation and the integration of empirical data into an agent-based model important. Empirical data can be used as input data for the model (to specify and calibrate the model at the micro level) and to test it (validate the simulation results at the macro level) [8,12]. In our case, empirical data was used for both purposes, i.e., to specify and validate the model.

In a survey by Heath et al. [5], they found that the majority of agent-based models is not validated both conceptually and operationally, which they deemed unacceptable. However, they also revealed that, over the 10-year evaluation period, there is a clear trend towards more validation efforts. More recent literature [7, 13] indicates that the situation has only been changing slowly since 2009.

In this paper, we present an agent-based model for which empirical data was collected from several sources and divided into two sets: data for model development and data for model validation. The model is intended to represent the wood markets in Switzerland. These markets have several peculiarities which qualify ABM as an appropriate modeling method. It was created to facilitate a better understanding of these markets by simulating scenarios focused on wood availability and allocation. An initial version of this model was presented in Kostadinov et al. [14], in which three main opportunities were identified to improve the model, namely the gathering of empirical data for the decision-making process of the agents, a more realistic modeling of wood transport routes (which affects transportation costs), and a better handling of the model boundaries (avoiding boundary effects). These issues are addressed in the present article. The model has been substantially redesigned and re-implemented to be more realistic with regard to these issues, while also improving the software architecture to reduce the model's execution time. The approach is demonstrated with an ex-post case study on the market entry of a bulk purchaser.

The following section gives an overview of the model, the methods applied, and the empirical base of the model. In section 3, results are presented and discussed. Section 4 concludes the article.

## 2 Materials and methods

In this section, first, a condensed description of the model is presented. Then, an overview of the applied calibration and validation methods is given. Finally, the empirical data used to calibrate and validate the model are described, including official statistics, data from our own surveys, and the historical event of a bulk purchaser entering the market in 2007 and becoming insolvent in 2010.

### 2.1 Description of the model

The following model description is based on the structure of the first sections of the ODD+D protocol [15], an extension of the ODD protocol [16,17]. The aim of ODD+D is to provide a better understanding of how human decision-making is modeled. This description should provide the reader with a basic understanding of the model, which is necessary to understand the subsequent chapters. An earlier version of the model is described in Holm et al. [18]; thus, parts of the model description may overlap.

#### 2.1.1 Purpose

The overarching goal of this study is to show ways how additional amounts of different wood assortments can be made available to consumers, as the sustainable potential of wood as a resource is currently not reached in the study region (the canton of Grisons (GR) in Switzerland), i.e., the annual growth of wood is larger than the annual amount harvested. The model was developed to provide insights into the processes of resource allocation in the modeled markets. It should help to identify the conditions under which resource availability can be increased, with a focus on the decision behavior of the agents and structural parameters, such as the presence of intermediaries.

The current version of the model is designed to be used by the authors to simulate scenarios on behalf of stakeholders. A direct operation of the model by the stakeholders is not intended owing to the complexity of the model.

#### 2.1.2 Entities, state variables, and scales

**Fig 1:**
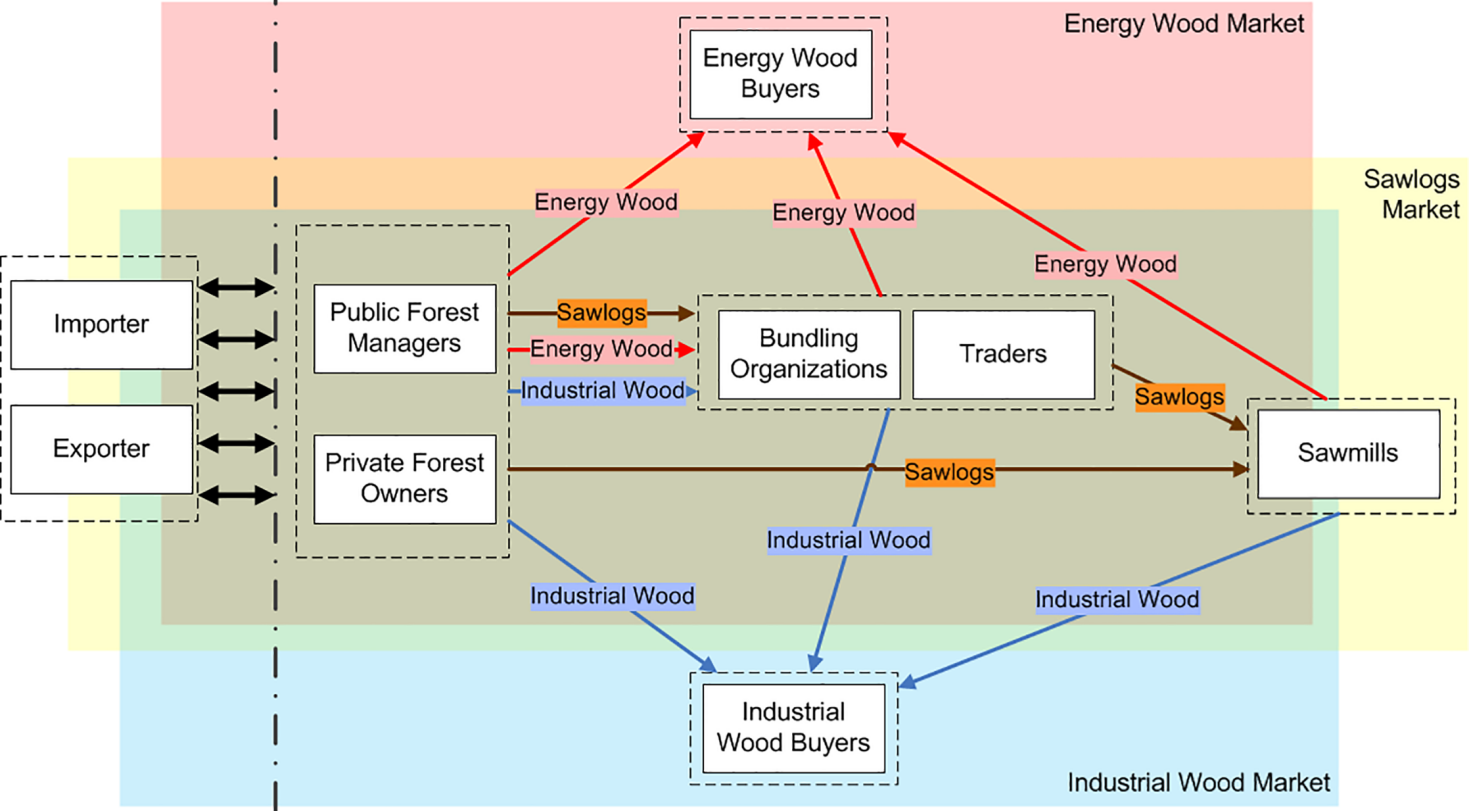
Conceptual model: Agents and markets.

The model consists of the following overlapping markets: the markets for sawlogs, which are the main product, and the markets for two side-products, namely industrial wood and energy wood. For each product, there is one market for softwood and another for hardwood, resulting in six markets in total. There are producing agents, intermediaries, and consumers for each of the products (see Fig 1). A typical model run simulates a 20-year period, where a single time-step represents one month.

**Fig 2:**
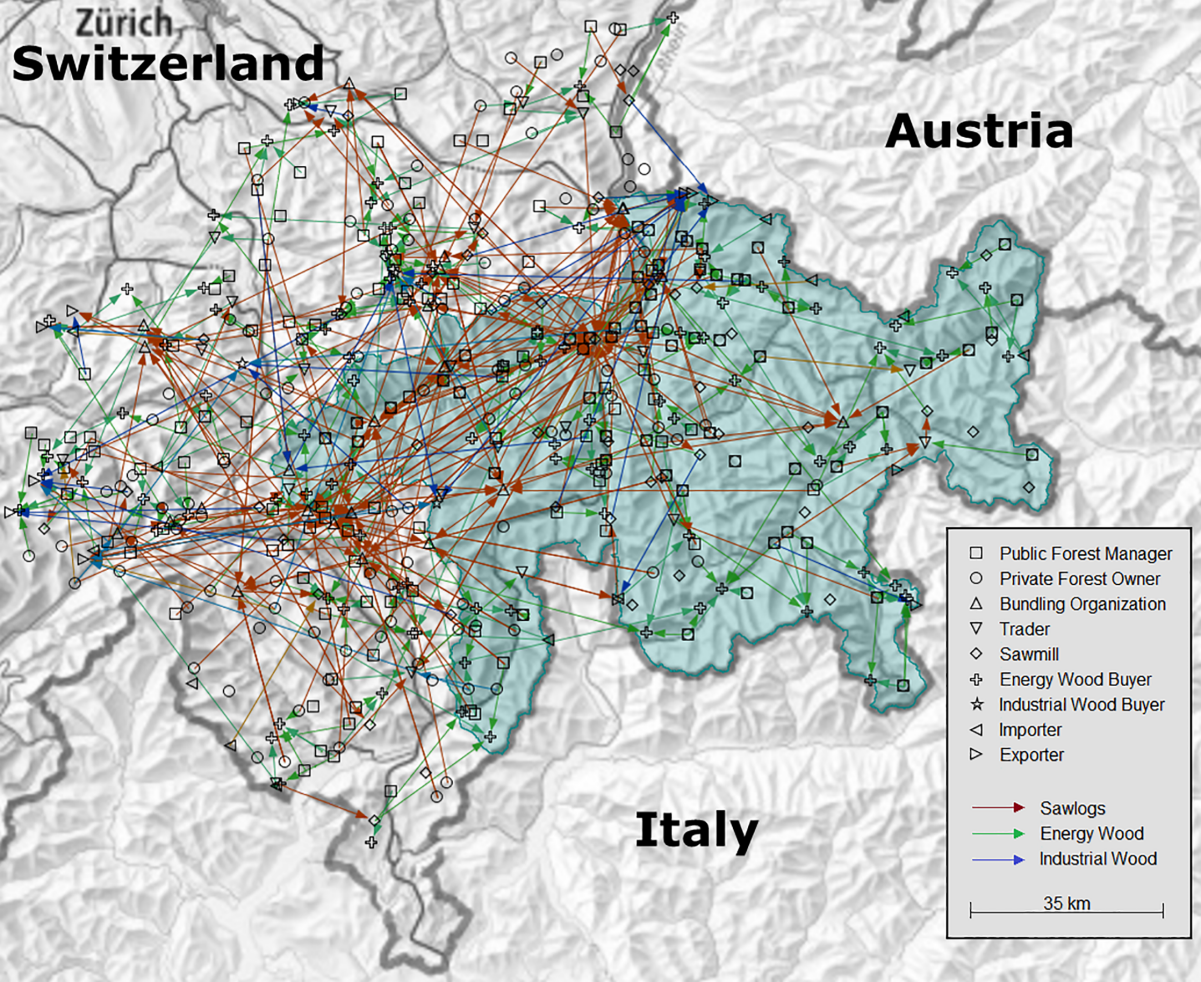
Map showing trading relations at one point in time. The colored area represents the study region (inner zone); nodes and arrows represent agents and deliveries, respectively.

As the model represents an existing geographical region, it is necessary to handle boundary effects (sometimes called border effects), which is a challenge in many spatial agent-based models. If artificial regions are used, such effects are often avoided by applying a torus ("doughnut") structure (e.g. [19,20,21]). However, in this case, the modeled region is real and highly dependent on adjacent areas, especially concerning the prices of wood, which depend on the global market prices; these are exogenous factors in the model. On its eastern side, the study region borders on other countries (with a different currency), whereas the western side of the study region borders on domestic regions. Therefore, we have two kinds of borders, which need to be handled differently. Where the study region borders on other countries, importer and exporter agents are distributed along the border to sell or buy wood at prices based on historical price data from adjacent countries, and the corresponding exchange rate. Where the study region borders on domestic regions, an additional belt of agents is modeled. These represent the part of the domestic market with a direct influence on the study region. We call this belt the outer zone of the model, while the study region itself is called the inner zone of the model. The agent quantities, properties, and their behavior are similar in both zones. The outer zone acts as a buffer zone to avoid boundary effects in the inner zone. This allows the evaluation of variables such as transportation distances in the inner zone. Consequently, the validation focused on the individual and the aggregate behavior of the agents in the inner zone. However, necessary parameter changes identified during calibration and validation were always applied for the agents in both the inner and the outer zone. For the evaluation of simulation results, only the agents in the inner zone are considered (Fig 2). With this approach, we managed to overcome the boundary problems we were facing in a previous study [14], which was one of the main issues identified therein.

Each agent has a fixed geographical position on the map that is assigned at the beginning of the simulation run. For public forest managers, this position corresponds to the real-world position of the agent in our study region. The positions of the other agent types are assigned randomly. The agent quantities are listed in Table 1. They reflect the actual number of market participants in the study region, unless they are marked as “aggregated”, which means that a single agent represents multiple real-world market participants. The following agent types exist in the model:

- **Public forest managers**: These agents manage the public forests in their area. In our study region, 88% of the forest is under public ownership [22], which makes them the most important agent group on the supply side of the markets. They sell wood of all six assortments.
- **Private forest owners**: In our study region, 8% of the forest is under private ownership [22] (the remaining 3.5% of the forest in the study region is hybrid property). In absolute numbers, there are 10'110 private forest owners in the study region that own a total forest area of 16'517 ha [22]. With an average size of 1.65 ha per private forest owner, the wood is usually not harvested by the owners themselves, but with the help of public forest managers or contractors. They are often mentored by a public forest manager. In the model, these agents are aggregated so that there is only one private forest owner agent in the territory of each public forest manager, representing (for model simplicity) the aggregate of all private owners in this territory. They sell wood of all six assortments.
- **Traders**: Traders buy all of the six wood assortments in the model, and try to sell them on the markets at a profit.
- **Bundling organizations**: These agents are cooperatives of small suppliers (private and public), structured to reduce distribution costs and increase market power. They are modeled as intermediaries that are tightly coupled to the affiliated suppliers.
- **Sawmills**: They buy sawlogs and process them into different wood products (for which the downstream markets are not included in the model). During the processing of sawlogs, residuals (tree bark, woodchips, shavings, and sawdust) are accumulated as byproducts and either used by the sawmill itself or sold on the market as energy wood and industrial wood.
- **Industrial wood buyers**: They buy industrial wood and process it into products such as pulp and paper. Downstream markets are not included in the model.
- **Energy wood buyers**: They buy energy wood, predominantly for heating purposes. This includes all consumers from single-family homes with a fireside, up to district heating distributors. These market participants are modeled as aggregated agents.
- **Importers**: They import wood from the outside to the inside of the modeled region.
- **Exporters**: They export wood from the inside to the outside of the modeled region.

**Table 1:** Quantity structure of the modeled agents.

Agent type	Number of agents (inner zone + outer zone)	Annual supply and/or demand per agent
Public Forest Managers	85 + 85	Annual maximum supply on average ca. 3500 m^3^ wood, thereof ca. 97% softwood. Distribution of supply values and geographical position reflect actual values in the study region. Softwood: 81% is provided as sawlogs; 13% as energy wood; 6% as industrial wood. Hardwood: 2% is provided as sawlogs; 95% as energy wood; 3% as industrial wood. These values can change over time, depending on assortment prices.
Private Forest Owners	85 + 85 (aggregated)	Annual maximum supply on average ca. 100 m^3^ wood, thereof ca. 60% softwood. Distribution of supply values and geographical position reflect actual values in the study region. Softwood: 81% is provided as sawlogs; 15% as energy wood; 4% as industrial wood. Hardwood: 1% is provided as sawlogs; 96% as energy wood; 3% as industrial wood. These values can change over time, depending on assortment prices.
Traders	12 + 12	Variable (try to buy and resell as much as possible)
Bundling Organizations	8 + 15	Variable (try to buy and resell as much as possible, but buy only from affiliated wood suppliers)
Sawmills	25 + 25	All sawmills process softwood, between 800 m^3^ and 8000 m^3^ (avg. ca. 2300 m^3^). Three sawmills each process 180 m^3^ hardwood in addition. Market entry or exit is possible.
Industrial Wood Buyers	1 + 2	Fixed demand of industrial wood: 4800 m^3^ softwood and 1200 m^3^ hardwood
Energy Wood Buyers	50 + 50 (aggregated)	Fixed demand of energy wood: 900 m^3^ softwood and 225 m^3^ hardwood
Importers	6 + 6	Sold amounts are theoretically unlimited, but annual increase is limited
Exporters	6 + 6	Bought amounts are theoretically unlimited, but annual increase is limited

#### 2.1.3 Process overview and scheduling

Box 1 shows the pseudocode [23] of the model’s main method. The six markets are executed consecutively, month after month, for a simulation period of 20 years. After the execution of each month, multiple evaluator classes analyze the current simulation state and write it to a file.

**Box 1. Pseudocode of the main method**

Simulation.start() {

    FOR EACH month {//20 years are simulated

        FOR EACH market {//six markets

            market.executeRound()

        }

        FOR EACH evaluator {//multiple evaluators monitor the simulation state

            evaluator.evaluteRound()

        }

    }

}

The execution of a single market round (one month) is depicted in Box 2. The most important step is the first, in which each agent has the possibility to conclude new contracts, either for the current month or for a forthcoming month. Thereby, the agents consider their current and forthcoming demand for or supply of a product, the stock, and the contracts that have already been concluded. The goal of each agent is to be able to meet the demand continuously; or, in the case of a wood supplier, to harvest and sell the wood equably during the harvesting months. As contracting parties, he prefers agents he already knows from successful transactions in the past.

**Box 2. Pseudocode of a market round**

Market.executeRound() {

        allAgents.shuffle()

        //step 1: all market participants try to conclude new contracts

        FOR EACH marketParticipant {

                marketParticipant.makeNewContracts();

        }

        //step 2: sellers prepare the deliveries (e.g. timber harvesting)

        FOR EACH seller {

                seller.prepareDelivery();

        }

        //step 3: sellers deliver

        FOR EACH seller {

                seller.executeContracts();

        }

        //step 4: intermediaries deliver

        FOR EACH intermediary {

                intermediary.executeContracts();

        }

        //step 5: buyers process the deliveries

        FOR EACH buyer {

                buyer.processDelivery();

        }

}

**Fig 3:**
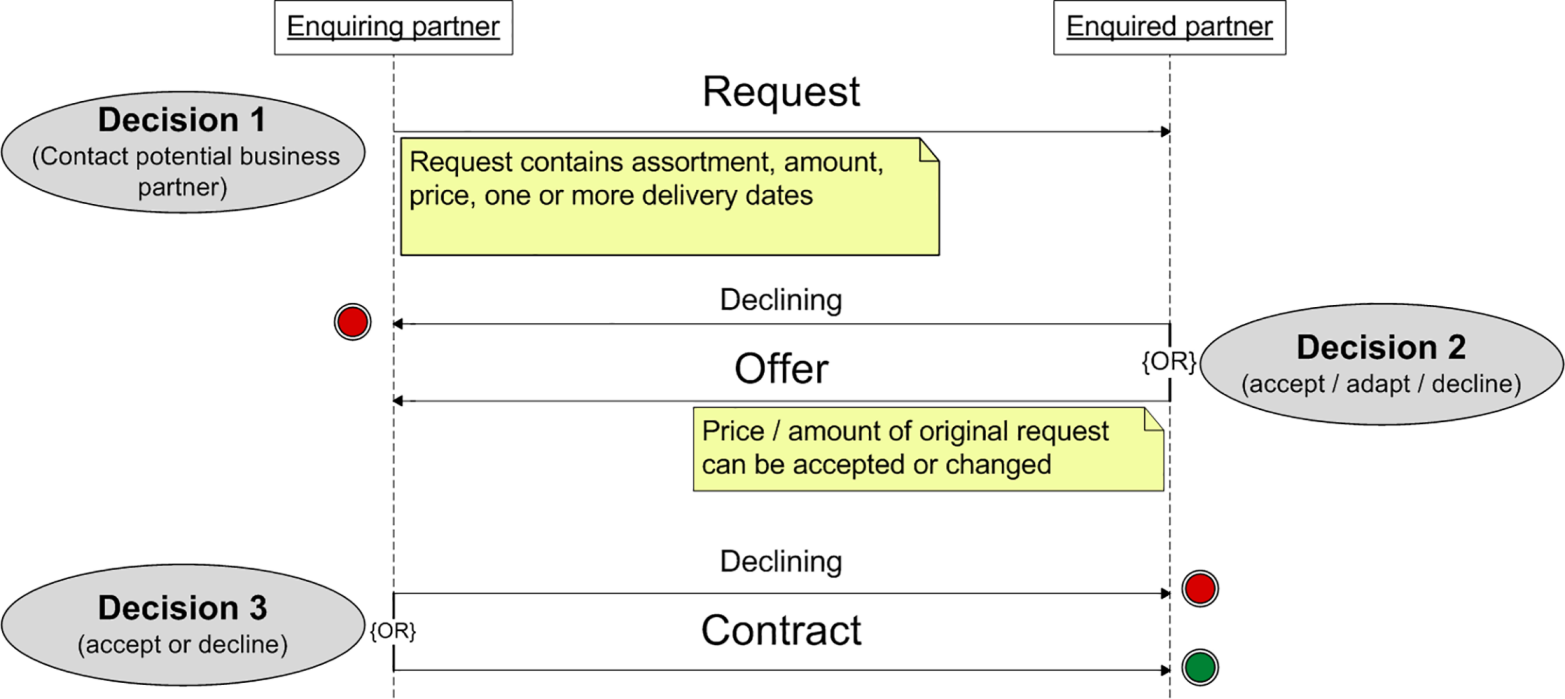
Conceptual model: Agent interaction. This diagram shows how agents conclude new contracts.

The core algorithm of interaction describes how two agents negotiate a new contract, and is illustrated in Fig 3; it is the same for all agents. The negotiation is initiated by an agent who wants to buy or sell wood from a certain assortment. The agent contacts a potential contract partner by sending him or her a request containing the assortment, amount, price, and delivery date. The contacted agent can either accept the request as-is, adapt the price and/or amount, or decline the request. In the first two cases, it is replied with an offer. The agent who initiated the negotiation then has a final opportunity to either accept or decline the offer (no further modifications of the offer are possible). If the agent accepts the offer, the contract is concluded, and will be executed on the specified delivery date(s). The decisions whether a request or offer should be accepted, adapted, or declined, is explained in the following section.

As opposed to the first version of the model [14], an agent does not have the possibility to compare several potential contracts and then choose the best one. When an agent receives a request or an offer from another agent, he decides immediately whether to accept or decline it (or to modify it, in certain cases). This approach was chosen because it reflects the common practice of the given market more realistically than the first approach. However, it implies special requirements in the decision algorithm, which are also explained in the next section.

Each agent has a list (herein, a “phonebook”) that contains potential contract partners in the surrounding area, with a trust value assigned to each contact. These trust values increase after successful negotiations and decrease after unsuccessful negotiations. They are an important criterion in the agents’ decision model. Among other things, contacts with a higher trust value have a higher chance of being considered when an agent wants to make a new contract.

#### 2.1.4 Theoretical background

As a contract is deliberately not concluded by selecting the best of several options, but by assessing them individually, each agent requires a function to evaluate a single potential contract. We use the following utility function, which is based on random utility theory [24], to allow our agents to decide whether a request or an offer is acceptable or not; this function is the basis of the agents' decision model:

$$U=\sum_{i=1}^{n}(\beta_{i}c_{i})+\varepsilon -\beta_{0}$$ Eq. 1

where *U* is the total utility of the request or offer, *n* is the number of decision criteria an agent considers in a decision situation, *β*_*i*_ is the part-worth utility of criterion *i*, *c*_*i*_ is the numerical value of criterion *i*, *ε* is a random component reflecting non-measurable factors in a person's decision, and *β*_*0*_ is the minimum utility required for a request or offer to be acceptable. A request or offer is accepted if the total utility is greater than zero.

The decision criteria *c*_*i*_ to *c*_*n*_ used by each agent group were defined in interviews and workshops. Then, the part-worth utilities were elicited in discrete choice experiments (DCE), a preference elicitation method widely used in marketing, as well as in other fields of economics. The suitability of using DCEs to parameterize the agents' decision model and the details of this approach are demonstrated in Holm et al. [18]. For the evaluation of the DCEs, we used the Hierarchical Bayes (HB) method, which calculates individual part-worth utilities for each subject, and is, therefore, most suitable for the agent-based paradigm. While the part-worth utilities for the criteria have been taken directly from the DCEs, *β*_*0*_ requires calibration (as a consequence of the experimental setup, where always three options are compared, which is usually not the case in reality). The random component ε is set to zero in the simulations presented here.

#### 2.1.5 Individual decision-making

Table 2 shows the objectives pursued by the agents and the decision criteria considered during contract negotiation.

**Table 2:** Objectives and decision criteria of the agents.

Agent type	Overall objectives	Decision criteria
Public Forest Managers and Private Forest Owners	Harvest the annual targeted amount, distributed as evenly as possible throughout the harvesting seasons, and sell the wood at a profit	Amount available (the annual cut is capped), amount in demand, trust in contract partner, margin (wood price minus harvesting costs)
Bundling Organizations	Bundle goods from the affiliated suppliers and sell at a profit	Sufficient margin
Traders	Buy and sell as much as possible at a profit	Price, trust in contract partner
Sawmills	Constant degree of capacity utilization throughout the year.	Buying (sawlogs): urgency, size of order, trust in supplier, price. Selling (by-products): utilized stock capacity, price, trust in buyer
Energy Wood Buyers	Covered demand during heating period	Urgency, price, trust in seller
Industrial Wood Buyers	Covered demand throughout the year	Urgency, price, trust in seller
Importers	Sell at international market price	Price
Exporters	Buy at international market price	Price

### 2.2 Model calibration and validation methods

#### 2.2.1 Overview

The goal of validation is to determine if the model is a sufficiently adequate representation of the real system. The validity of a model should be determined with respect to its purpose [25]. The main purpose of our model is to investigate resource availability and resource allocation under conditions defined by the model user. Therefore, the most important variables in the validation process are the provided amounts and prices. There are different concepts of validity [26]; here, we focus on empirical validity, i.e. the "validity of a model with respect to [empirical] data" [27].

Two basic aspects of a model that need to be validated are the conceptual model (conceptual validity) and the simulation output (operational validity) [5,25]. In addition, some authors mention (program) verification as a part of model validation, i.e., measures to ensure that the computer model is a correct implementation of the conceptual model [25,28]; and, likewise, data validity, i.e., obtaining and using adequate and correct data [25]. Our conceptual model was validated in several workshops with stakeholders during the model-building process, which started by conducting open interviews with real persons corresponding to the model agents, followed by surveys with more specific questions and a larger target group. The simulation output was validated mainly by comparing it to historical observations and data from our own surveys, and also by checking its consistency with expert knowledge. This part of the validation is explained in more detail in subsequent sections. For program verification, standard software testing approaches, such as assertions and unit-tests, were applied. As missing (or low-quality) empirical data is one of the main problems in the validation process [27,29], we attempted to ensure data validity by conducting our own tailored surveys, which are described in detail in section 2.3.2.

A further distinction can be made concerning the type of validity [30]:

- Replicative validity: the model can reproduce known behavior of the real system.
- Predictive Validity: the model can predict system behavior that is not yet known.
- Structural validity: the model internally behaves similarly to the real system.

Zeigler specifies these three types of validity as building on each other, with replicative validity at the lowest and structural validity at the highest level. However, in social sciences, there are also models that attempt to be structurally valid without regarding replicative or predictive validity [31]; from this point of view, these three types of validity do not necessarily depend on each other. Since our main goal is to understand the processes of resource availability and resource allocation, we aim at replicative and structural validity. For the former, we validated amounts and prices on an aggregated level. For the latter, we looked at variables concerning the individual level, such as behavioral variables and variables characterizing the structure of interaction. These were validated by comparing them to the data gathered in our own surveys. This type of empirical data and knowledge regarding micro-level phenomena is indispensable to understand the causal mechanisms of the processes under study [12].

Obviously, it is impossible to gather empirical data for all individual micro-level variables in the model; thus, parameterization and calibration were used in addition. According to Railsback & Grimm [32], parameterization is the process of selecting values for the input parameters of the model. Calibration is a special case of parameterization where values for important parameters are set in such a way that the model reproduces patterns observed in the real system. The purpose of calibration is either to fine-tune known parameters (direct calibration) or to estimate values for parameters with completely unknown values (indirect calibration) [32,33]. From a formal point of view, calibration is an optimization problem [29]. A third purpose of calibration is to determine whether the model is able to reproduce an expected aggregate behavior by adjusting the input parameters; because, if not, its structure might not be sufficiently realistic [32]. As structural validity is one of our requirements, this is an important measure to recognize whether our model needs further improvement or is already sufficiently realistic for the given purpose. The reproduction of patterns observed in the real system is also referred to as "pattern-oriented modeling" (POM), especially in ecology [34,35]. POM aims at improving the structural validity by finding a model structure and model parameters that reproduce multiple patterns simultaneously. The observed patterns preferably occur on different levels of aggregation: in a market model such as the one presented here, a pattern on a high level of aggregation could be traded quantities in a certain region over time, on a lower level of aggregation the typical delivery quantity of a single transaction.

According to the definition of prediction used by Kelly et al. [4], we also aim at predictive validity in the sense that the model must be able to estimate the system behavior when exogenous model variables are changed, so that their influence on the system behavior can be examined. There is a long-standing controversy regarding whether prediction and explanation are equal [31,36,37]. Some authors also state that "prediction should be the real aim of every model" [38] or that "validation of social simulation models requires prediction" [39]. In contrast, they are seen as different by other authors, such as Epstein [3], who illustrates the distinction with the example that earthquakes are explainable, but not predictable. As stated in the introduction, we follow the definition of Kelly et al. [4] in this paper.

#### 2.2.2 Validation techniques applied

An overview of validation techniques is given by Sargent [25]. We used the following for the validation of our model:

- **Animation**: A map showing the development of the agents' trading relations over time was observed during simulation (cf. Fig 2), as well as the resource flows among agents of different types.
- **Event Validity**: The behavior of the model after a market entry of a very large sawmill agent was compared to such an event that was observed in the real system some years ago (details will be presented in section 2.3.3).
- **Face Validity**: The behavior of the model (as well as a presentation of the conceptual model) was discussed with domain experts.
- **Historical Data Validation:** Historical data on amounts and prices were used to validate the model. This will be explained in more detail in section 2.3.1.
- **Operational Graphics**: A vast number of variables were observed during simulation at different levels of aggregation: the most important variables were observed at the level of individual agents; others were aggregated over all agents or agents of some type. It was observed, for example, whether all agents were sufficiently supplied, and whether local price differences stayed in a realistic range.
- **Parameter Variability-Sensitivity Analysis**: This was conducted together with the calibration of the model to determine the effect of the input parameters on the simulation results.
- **Traces**: A separate application program was developed to trace individual agents in more detail. For every agent type, a few agents were selected for which a snapshot of each simulation time step was recorded during the simulation. Such a snapshot includes an agent's current stock of all resources and the current status of all negotiations with other agents. These snapshots were then analyzed with this tracing application in a post-processing step. This approach allows to examine in detail which negotiations led to a contract and which not, and reveals the reasons for the underlying decisions. It also shows the activity of an agent, i.e. how many other agents are contacted, and how many negotiations are initiated from other agents. The tracing application thereby not only allows validation from the perspective of single agents; it is a very helpful instrument in all stages of model development, as it also facilitates verification (in particular finding and fixing bugs) and supports the in-depth analysis of emerging phenomena.

Some of these techniques can be realized with statistical tests (e.g. hypothesis testing); others only with non-statistical approaches that involve subjective judgments, e.g., by expert opinion or qualitative comparisons [5,25]. However, in almost all cases related to agent-based modeling, they are applied non-statistically [5]. We also focused on expert opinion and qualitative comparisons here.

There are two further aspects worth mentioning. The first is the selection of the validation period, i.e., the years over which the empirical data is compared to the simulated data (cf. [27]). We started in the years between 2001 and 2004 (depending on the variable) for the following reasons: first, there was a hurricane in 1999 which felled trees in the volume of approximately three times the annual cut in Switzerland [40], which had a strong impact on the market. The second reason is the lack of data availability or quality prior to these years. Third, our simulations start in 2001, and the model needs several time-steps to settle down (relationships between agents need to be established etc.); therefore, the initial simulation months cannot be used for validation, as they might be biased.

The second aspect is the determination of when to stop the validation (and, thereby, the related calibration process). As structural validity is one of our goals, it would be inaccurate to attempt to improve the empirical validity of the model by evaluating solely the macro-behavior, thereby calibrating the input parameters to unrealistic values [41]. Therefore, we followed the approach of validating until every validation variable (on micro and macro level) was either in a realistic range or its difference was explainable (and acceptable for the model purpose).

### 2.3 Empirical data for calibration and validation

According to Kelly et al. [4], "Predictive models are generally required to have some level of accuracy in reproducing historic observations, and thus require data for calibration, and other independent data for validation.". In the following, we present the empirical data used in these two processes, and how these data were used.

#### 2.3.1 Data from the Swiss Federal Statistical Office

A wide range of fine-grained data on the wood markets in Switzerland is provided by the Swiss Federal Statistical Office (FSO). The most valuable data for our model regards the amounts of harvested and processed wood, and the prices thereof. The following paragraphs provide an overview of these data and show how we prioritized them to validate our model.

For each of the six assortments represented in the model, data on the yearly harvested amount from 2004 until 2014 per forest owner type (public or private) in our study region, canton GR, is available. This gives us 12 values per year to use for the validation. Depending on the importance of the assortment in the study region, different priorities were assigned to them, while some even were omitted (Table 3). Finally, the amounts of wood processed by sawmills in the years 2002, 2007, and 2012 in our study region were used for the validation of the model (this data is only available in 5-year increments). Here, softwood is considered to be of high priority, while hardwood is considered to be of low priority as it constitutes less than 0.5% of the total amount processed in the study region.

**Table 3:** Data for harvested wood available for validation.

Forest property type	Assortment	Avg. m^3^/a produced 2004–2014	Coefficient of variation (σ/μ) 2004–2014	Validation priority
Public	Sawlogs softwood	249’097	9.6%	high
	Sawlogs hardwood	311	79.7%	low
	Energy wood softwood	65’747	27.8%	high
	Energy wood hardwood	14’130	25.7%	high
	Industrial wood softwood	7’492	13.9%	medium
	Industrial wood hardwood	328	117.0%	*omitted*
Private	Sawlogs softwood	21’089	39.5%	high
	Sawlogs hardwood	126	176.5%	*omitted*
	Energy wood softwood	5’779	48.0%	medium
	Energy wood hardwood	4’318	20.1%	medium
	Industrial wood softwood	538	45.7%	low
	Industrial wood hardwood	200	139.1%	*omitted*

Each row represents an assortment and thus a variable for which a time series exists for model validation. The averages and coefficients of variation (CV) are shown to indicate the relevance of the variable in the validation process. Assortments with small annual amounts (below 1000 m^3^) are considered low priority. If there is a high variation in addition, the assortment is omitted from the validation

Price data for all six simulated assortments were used for validation. This data is available on a quarterly basis from 2001 to 2014. The validation priorities are based on these for the amounts (Table 3): prices for sawlogs (softwood) and energy wood (softwood and hardwood) are considered high priority; industrial wood (softwood) medium priority; the rest is low priority.

#### 2.3.2 Data from own surveys

Six surveys were conducted to obtain detailed insights into the market participants’ behavior and the market structure. The survey participants were informed that their answers to the questions in the questionnaire will be used for this research project, in an anonymized form. Table 4 gives an overview of these surveys: the four most important agent types in our model were surveyed, whereas the others have been built based on expert knowledge. The key agents are the public forest managers, as they manage the biggest part of the forest area (70% in the whole country, 88% in our main study region of canton GR [22]), while also providing advice to private forest owners; therefore, they have the main control of the wood supply. They were surveyed in a full population survey in three different regions. Because of the peculiarities of these regions, different results for each region were expected and confirmed empirically. The respondent rate of this agent group was high (approximately 70–75%). The public forest manager survey in the regions AG (canton of Aargau) and GR (canton of Grisons) were completed on paper as an additional agenda item on the semiannual public forest manager meetings, where most of the public forest managers of the corresponding region were present. These meetings took place in March and April 2014. For the region BE (canton of Bern), a mail containing a link to the online survey was sent to all public forest managers in the region. This survey was online in December 2015.

**Table 4:** Overview of conducted surveys.

	Region	N	n	Year	DCE included
***Suppliers***					
Public Forest Managers	AG	ca. 80	55	2014	yes
Public Forest Managers	GR	ca. 90	68	2014	yes
Public Forest Managers	BE	ca. 100	77	2015	yes
Private Forest Owner	BE	ca. 36’000 (contacted: 1’440)	69	2016	no
***Demanders***					
Sawmill Operators	CH	ca. 400	21	2015	yes
Energy Wood Buyers	CH	ca. 2000 (contacted: 744^a^)	112	2016	yes

Regions AG, GR, and BE correspond to cantons in Switzerland; CH corresponds to Switzerland as a whole. The last column states whether a discrete choice experiment (DCE) was included in the survey.

^a^ 744 public forest managers were contacted and asked to forward the survey to their main energy wood buyer.

The survey participants in the private forest owner survey were recruited in March 2016 by sending them a letter with a link to an online survey. In the this survey, the response rate was low (4.8%). The answers revealed that those responding seem to have a very strong relation to their forest, and this is, according to expert opinion, a minority in Switzerland. Thus, the survey results are highly likely to have a strong sample selection bias [42]. The results of this survey were, therefore, omitted from the use in the model.

The sawmill operators survey was sent by e-mail as a pdf form to the members of the Swiss association of the timber industry in April 2015. While the response rate of this survey appears rather low at first glance (5.25%), our sample covers 41% of the countrywide processing capacity. This can be explained by the power-law distribution of the sawmill sizes. In 2014, approximately 1.87 million m^3^ of sawlogs were cut in Switzerland [43]. Approximately one third of this was processed in sawmills with an annual cut below 10'000 m^3^, one third between 10'000 m^3^ and 100'000 m^3^, and one third above 100'000 m^3^. We cover 11% of the processed quantity of the first class, 14% of the second class, and 100% of the class with the largest sawmills.

The energy wood buyers had to be contacted indirectly via public forest managers. A letter was sent to them in January 2016 and they were asked to forward a second letter with a link to the survey to their main energy wood buyer. This approach obviously already reduced the number of energy wood buyers that received the survey, but was the only possibility to get in contact with the energy wood buyers. However, the data quality of the 112 answered surveys was good and the survey provided valuable data for the model.

In the following paragraphs, we present which study results were used for which purpose in the model; some were used for model calibration, while others were used as validation data. Whenever we assumed that the model could predict a behavior that could potentially be falsified by a survey result, we used this survey result as validation data. For a few variables, only the average (over all agents) was validated; for most others, the distribution was also included by taking the interquartile range (IQR) into account, i.e., the range in which 50% of the values lie. The consideration of the IQR as an additional measure aims at improving the confidence in the model, as averages alone do not provide information about the variation, and even can be misleading if the underlying distribution is skewed.

**Public forest manager surveys:** From the three public forest manager surveys conducted, mainly the results from the study in canton GR were integrated into the model. While canton AG is flat terrain, canton GR is mountainous, which leads to large differences in these wood markets (e.g., owing to different harvesting costs). Therefore, differences in the results of these two surveys were used to identify parts of the model that need to be parameterizable, so that the model can be used in the future to simulate different regions. The survey in canton BE contained an additional section where public forest managers were asked questions regarding their mentoring of private forest owners. These results were used to compensate for the inapplicable private forest owner survey. Table 5 gives an overview of the results relevant to the model, and how they were used.

**Table 5:** Survey results from the public forest manager surveys and their use in the model.

Survey element	Use	Details
Discrete Choice Experiment	Input / Calibration	Basis of the decision model of the public forest manager agents and private forest owner agents.
Percentage of wood reserved for regular customers (not bound by contract)	Input / Calibration	This variable is important for the conclusion of contracts between business partners with no prior knowledge of each other. The following averages were used: sawlogs: 42%, energy wood: 55%, industrial wood: 25%
Own consumption of private forest owners per assortment	Input / Calibration	Averages used: sawlogs: 10%, Energy wood: 60%, industrial wood: 5%
Number of incoming requests per year (per assortment)	Validation	Averages (IQR in brackets): sawlogs: 5 (2–9), energy wood: 12 (1–20), industrial wood: 1 (0–2)
Percentage of incoming requests per year that were rejected (per assortment)	Validation	Averages (IQR in brackets): sawlogs: 25% (0–40%), energy wood: 20% (0–40%), industrial wood: 30% (0–50%)

**Sawmill operators survey**: The data from the sawmill operators survey and their use in the model are listed in Table 6. Some of the results are used as stylized facts (cf. [8]).

**Table 6:** Survey results from the sawmill operators survey and their use in the model.

Survey element	Use	Details
Discrete Choice Experiment	Input / Calibration	Basis of the decision model of the sawmill agents
Stock capacity	Input / Calibration	A full warehouse covers the demand for two months.
Utilized stock capacity	Validation	64% on average
Duration of business relationships	Validation	Stylized fact: business relationships are usually long-term (>10 years).
Percentage of transportation costs in relation to the total costs per purchased m^3^.	Validation	Average 15%, IQR 12–17%.
Supply perimeter (distance between plant and forest where >90% of the wood is sourced).	Validation	Average 43 km, IQR 25–50 km
Number of incoming requests per year	Validation	Average 25, IQR 6–43
Number of outgoing requests per year	Validation	Average 10, IQR 2–14
Percentage of annual delivery quantity per supplier type	Validation	Averages (IQR in brackets): Public Forest Managers: 42% (20–66%) Bundlers: 38% (6–52%) Traders: 20% (14–26%)
Annual delivery quantity of a single supplier per type (the amount *one* sawmill obtains from *one* supplier)	Validation	Averages (IQR in brackets): Public Forest Managers: 600 m^3^ (250–950 m^3^) Bundlers: 3700 m^3^ (1063–5600 m^3^) Traders: 1150 m^3^ (400–1570 m^3^)

**Energy wood buyers survey**: Table 7 gives an overview of the energy wood buyers survey results and their use in the model.

**Table 7:** Survey results from the energy wood buyers survey and their use in the model.

Survey element	Use	Details
Discrete Choice Experiment	Input / Calibration	Basis of the decision model of the energy wood buyer agents
Contract duration	Input / Calibration	Usually 5 to 15 years (10 years on average)
Share of softwood in total wood amount processed	Input / Calibration	Study region: 85% softwood, 15% hardwood (whole country: 50% softwood, 50% hardwood)
Stock capacity	Input / Calibration	A full warehouse covers the demand for one month.
Duration of business relationships	Validation	Stylized fact: business relationships are usually long-term (87% >5 years, 60% >10 years)
Supply perimeter (distance between plant and forest where >90% of the wood is sourced).	Validation	Average 15 km, IQR 5–20 km
Imported amounts	Validation	Import of energy wood is very unusual
Number of incoming requests per year	Validation	Average 1.5
Number of outgoing requests per year	Validation	Average 1

#### 2.3.3 Case study

As a further validation step, we use the model in the context of a historical case of a very large sawmill entering the market in our study region and becoming insolvent only a few years afterwards. The sawmill was located in the Domat/Ems, a village in our study region located at a national highway, and the site also had direct access to the railways which should reduce transportation costs. The sawmill started operating in 2007, sawlogs were delivered to the site starting in October 2006 [44]. It was the largest sawmill ever built in Switzerland, having a processing capacity approximately three times higher than the previously largest sawmill. The sawmill had difficulties to purchase sufficient amounts of sawlogs to be profitable, which finally lead to its insolvency in 2010 [44]. Using this case as an additional validation step, we want to check whether the model is able to reproduce the fact that the sawmill was not able to obtain sufficient sawlogs to become profitable in the time that it was on the market.

## 3 Results and discussion

First, this section describes the results of the model validation with a focus on historical data validity (by comparing the model output to the empirical data presented in the method section) and event validity (by reproducing the historical event described in the case study). Then, additional insights gained by simulating the case study are presented. As the model is stochastic, all simulation results presented here represent the average of 100 runs.

### 3.1 Validation

#### 3.1.1 Amounts

**Fig 4:**
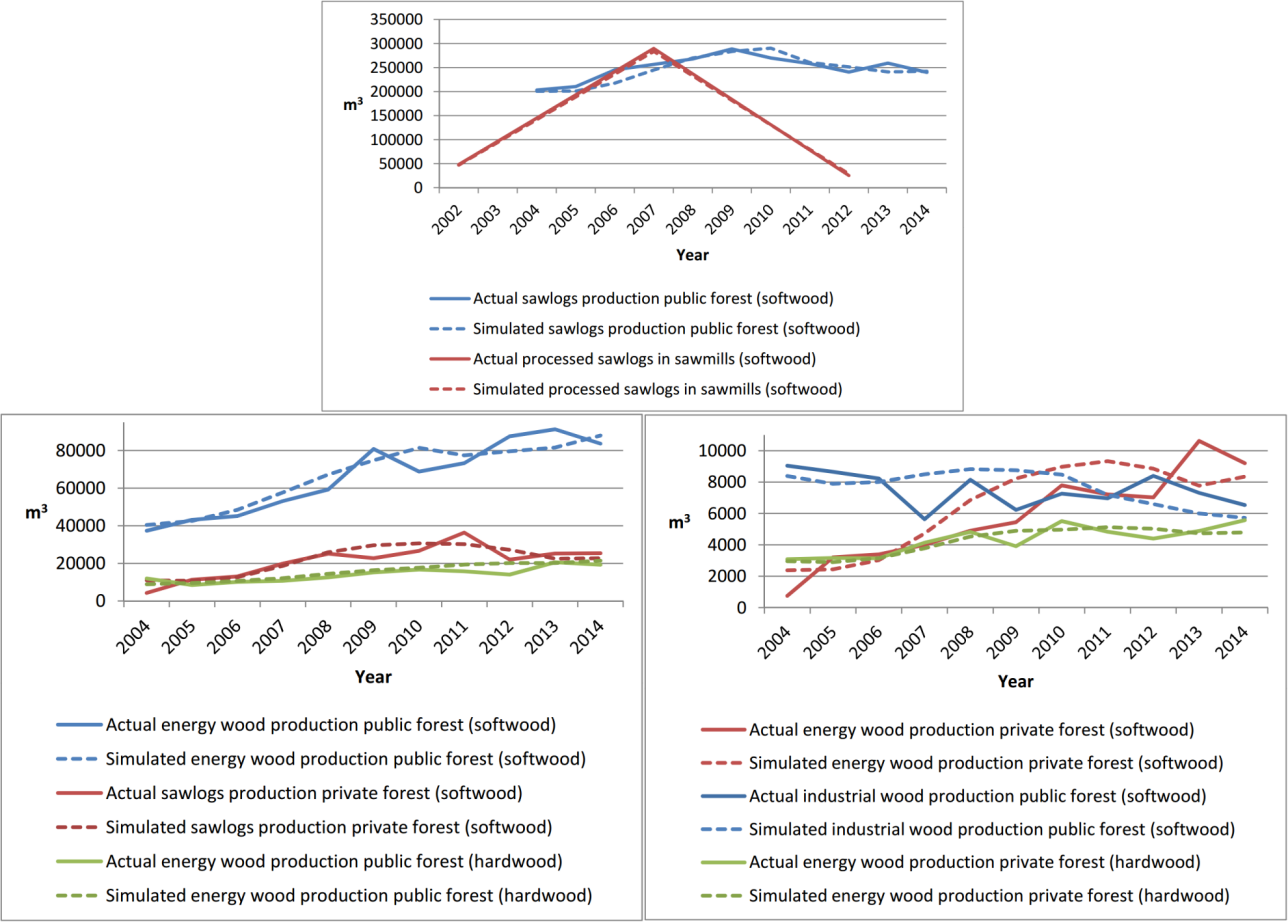
Comparison of actual historical and simulated data over time. The diagram at the top and at the bottom left show produced and processed amounts classified as high-priority for validation, and the diagram at the bottom right the processed amounts classified as medium priority. The diagrams show that the model is able to approximate the trends of produced and processed amounts in the specified validation period with a sufficient level of accuracy.

Fig 4 shows the simulated amounts produced and processed in comparison to the actual historical amounts for the assortments considered high or medium validation priority; the figures for the assortments considered low validation priority are shown in the appendix (Supporting information S1 Fig). The model is able to approximate the trends of the actual variable values over the evaluated period.

The main factors influencing wood production in the model are prices. Higher absolute prices increase the production by allowing wood harvesting in regions with higher harvesting costs, e.g., in mountainous terrain. The relative price levels of the different assortments shift the shares of the produced assortments (sawlogs, energy wood, and industrial wood). Private forest owners thereby have a wider scope than public forest managers, i.e., the shifting of the shares of the different assortments can be larger. These price elasticity parameters were not known and, therefore, needed to be calibrated indirectly (cf. section 2.2.1) to match the available empirical data regarding system behavior.

The top-left diagram in Fig 4 shows the processed amounts in the study region in the years 2002, 2007, and 2012, together with the harvested amounts from 2004 to 2014. The bulk purchaser analyzed in our case study was on the market from 2007 to 2010, which explains the processing peak in 2007. The differences between production and sawn wood in the years before and after also show why such a bulk purchaser was expected to mobilize more wood in the study region.

The validation results presented in Fig 4 show how closely the historical data can be approximated by the model. This is important for the requirement that the model must be able to show how wood availability can be increased. While price elasticity plays an important role therein, it is not the only factor: given the mountainous terrain of our study region with hardly-accessible areas, a higher production level is only possible by accepting higher harvesting costs, which again affects the decisions of the agents.

#### 3.1.2 Prices

**Fig 5:**
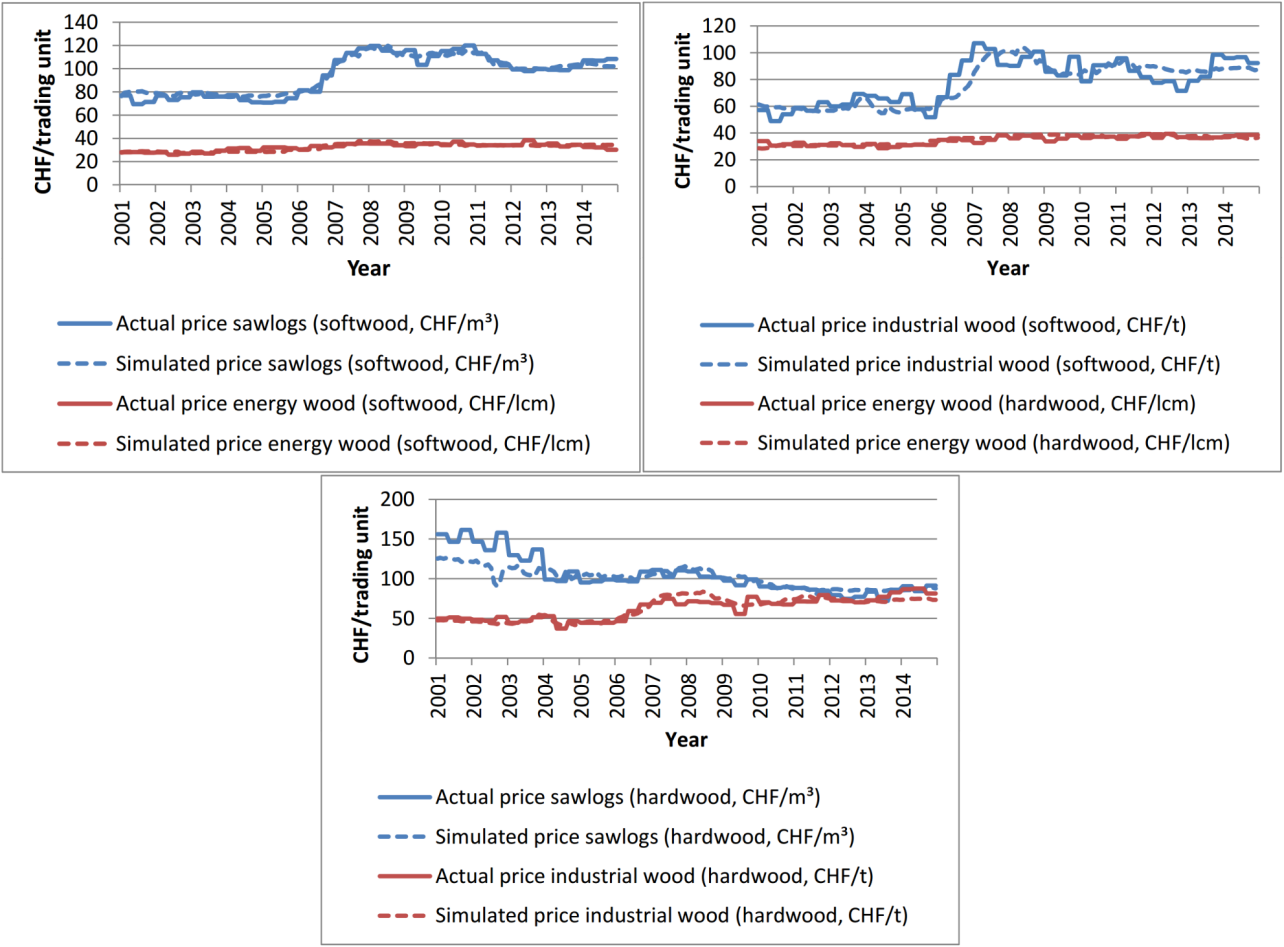
Simulated prices compared to the actual historical prices from 2001–2014. While the model internally always operates in m^3^, the prices are expressed here per trading unit, which depends on the assortment (lcm = loose cubic meters). In the first 2–3 years simulated, the model needs to settle, which explains the gaps between the actual and simulated values at the beginning of the simulation.

International wood prices and the exchange rate between the study region and adjacent countries are exogenous variables in the model, and the prices in the study region depend largely on international prices of the assortments. Therefore, it is a challenge for the model to reproduce local prices during periods when they differ from international prices. This was mainly the case around the time of the market presence of the bulk purchaser analyzed in the case study. The largest differences between local and international prices were observed for the most important assortment, sawlogs softwood. Fig 5 shows that the model is able to approximate the historical local prices of the six simulated assortments.

An important endogenous variable influencing the local prices on the supply side is the annual harvested amount, which influences harvesting costs and, thereby, the supply price. On the demand side, insufficient degrees of capacity utilization increase the willingness to pay and vice versa.

The ability of the model to reproduce local prices is relevant for the goal of understanding resource availability and allocation, as prices are a crucial factor in the decision model of every agent.

#### 3.1.3 Validation data from own surveys

Table 8 summarizes the extent to which the model was able to replicate the empirical data from the surveys presented in the method section. The majority of the results could be reproduced in an acceptable range; the reasons for larger discrepancies are explained. Validating the model with this empirical data is important because structural validity has a high relevance for our modeling purpose of system understanding, in particular, obtaining better insights into the processes of resource allocation. Averages and IQRs were calculated at each simulated time step over all agents of the concerned type. Finally, these values were averaged over the whole simulation period.

**Table 8:** Comparison of empirical data from surveys with simulation data.

Survey question	Values from surveys	Simulated values	Rating
	*(average*, *IQR in brackets when relevant)*		*(+*,*0*,*-)*
***Public Forest Manager Survey***			
Number of incoming requests per year (per assortment)	Sawlogs: 5 (2–9)	5.2 (2.4–7.4)	+
	Energy wood: 12 (1–20)	4.7 ^a^ (1.8–6.6)	
	Industrial wood: 1 (0–2)	0.4 (0–0.4)	
Percentage of incoming requests per year that were rejected (per assortment)	Sawlogs: 25% (0–40%)	57%, (28–94%)	- ^b^
	Energy wood: 20% (0–40%)	65%, (36–97%)	
	Industrial wood: 30% (0–50%)	45%, (6–85%)	
***Sawmill Survey***			
Utilized stock capacity	64%	77%	+
Duration of business relationships	Stylized fact: business relationships are usually long-term (>10 years).	Affirmed	
Transportation costs in relation to total costs per purchased m^3^	15% (12–17%).	16% (10–20%)	+
Supply Perimeter	43 km (25–50 km)	44 km (30–54 km)	+
Incoming Requests	25 (6–43)	27 (19–32)	+
Outgoing Requests	10 (2–14)	9.0 ^c^ (7.6–9.9)	+
Percentage of annual delivery quantity per supplier type	Public forest managers: 42% (20–66%)	45% (26–64%)	+
	Bundlers: 38% (6–52%)	37% (14–57%)	
	Traders: 20% (14–26%)	18% (4–27%)	
Annual delivery quantity of a single supplier per type ^d^	Public forest managers: 600 m^3^ (250–950 m^3^)	1982 m^3^	+ ^e^
	Bundlers: 3700 m^3^ (1063–5600 m^3^)	6550 m^3^	
	Traders: 1150 m^3^ (400–1570 m^3^)	1452 m^3^	
***Energy Wood Buyers Survey***			
Duration of business relationships	Stylized fact: business relationships are usually long-term (87% >5 years, 60% >10 years)	Affirmed	
Supply perimeter	15 km (5–20 km)	19 km (10–22 km)	+
Imported amounts	Import of energy wood is very unusual	8% is imported	0 ^f^
Incoming requests per year	1.5	12	- ^g^
Outgoing requests per year	1	18	- ^g^

^a^ Energy wood buyers are aggregated agents in the model, which may cause the discrepancy to the survey.

^b^ An explanation for this discrepancy is that, in reality, market participants might have a better sense of which public forest manager is the most promising for the next transaction. Calibrating the model for these variables was difficult: with data-mining techniques, heuristics were found and integrated into the agents’ decision model, which at least lowered the discrepancies to the empirical values.

^c^ For the calculation of the average (but not of the IQR), the bulk purchaser of the case study was excluded.

^d^ This variable was only evaluated for the bulk purchaser of the case study. Besides this large sawmill, there are only very small sawmills in the study region, which are on the one hand usually supplied by only a few suppliers, on the other hand underrepresented in our survey.

^e^ The values for bundlers and traders are around the upper limit of the IQR, which is acceptable. The value for public forest managers is approximately twice as high as the upper limit of the IQR. This can be explained by the forests in our study region GR, which consist of approximately 90% softwood. In contrast, the survey has been conducted over the whole of Switzerland, where forests consist of approximately 50% softwood. Therefore a typical public forest owner in GR has almost double the amount of softwood available, and softwood is what sawmills are mainly processing. This explanation was confirmed by simulations with the share of softwood set to 50%; then, the value for public forest managers was also around the upper limit of the IQR.

^f^ Approximately two thirds of the study region’s border is an international border; therefore, some border regions may import wood from the adjacent neighboring country.

^g^ Energy wood buyer agents are aggregated agents in the model and therefore represent multiple real-world buyers at all scales, whereas the survey participants were large-scale heating plant operators. They usually have one or a few long-term contracts, whereas smaller energy wood buyers may buy their energy wood as required.

#### 3.1.4 Case study

**Fig 6:**
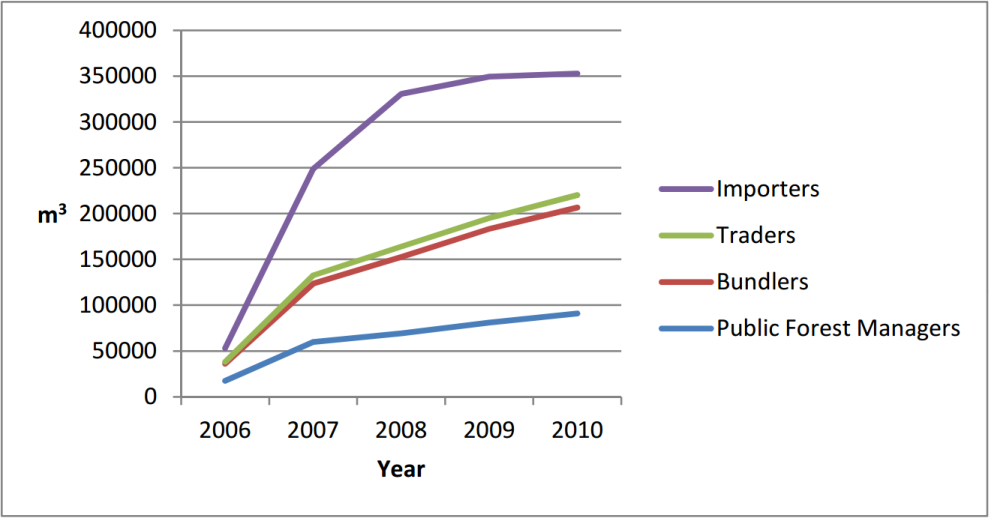
Stacked chart showing the simulated amounts supplied per supplier type for the sawmill under study. The capacity of the sawmill was approximately 800'000 m^3^ per year; therefore, the simulated degree of processing capacity utilization in 2010 was approximately 44%. Our surveys showed that large sawmills in Switzerland have a degree of capacity utilization of approximately 85% on average.

The model was able to reproduce the fact that the large-sized sawmill was not able to reach a profitable degree of capacity utilization during the time it was on the market. The simulated amounts supplied to the sawmill are shown in Fig 6.

The reasons why the sawmill was not able to purchase sufficient wood already became apparent during the model-building process. Our surveys showed that existing business relationships are relatively stable, and the majority of the annual harvested wood is already reserved for regular customers, even without contracts. Trust plays an important role in the Swiss wood markets [14]; therefore, wood suppliers are cautious regarding new contract partners and aim to preserve their business relationships with existing regular customers. Hence, a new market player first has to gain the wood suppliers' trust by buying low amounts and proving his reliability. With increasing trust, the new player will be able to buy increasing amounts of wood. This is a slow process, and is especially critical if the new player is a bulk purchaser that needs to process large amounts of sawlogs to be profitable.

Looking at the data of the produced amounts used for validation, an increase in wood production could be observed when this bulk purchaser became active in the market. The additionally harvested wood could have been supplied to the bulk purchaser, while still satisfying existing business relations. However, in reality, according to expert knowledge, this wood was mainly exported—this was also the case in our simulations.

### 3.2 Additional insights

Our simulations of the case study showed that this sawmill not only had difficulties in being supplied with sufficient amounts of wood, but was also required to pay approximately 9% more than its competitors on average. If the willingness to pay was reduced (by changing β_0_ in the decision behavior of the agent, i.e., the utility threshold for accepting an offer or rejecting it) so that the sawmill paid prices similar to those its competitors paid, the total amount supplied per year dropped to approximately 100'000 m^3^.

In our surveys, we observed that public forest managers have a certain percentage of sawlogs that they reserve for regular customers, even without a contract in place. This parameter has a value of 42% in our study region GR and is even higher (62%) in the two other regions surveyed, AG and BE. Surprisingly, reducing this value to zero does not change the sawmill's supply rate considerably, but lowers the supply prices that the sawmill is required to pay. A combination of several reasons may explain this observation: first, not reserving wood for regular customers does not prevent that wood from still being sold to these customers. Second, such reservations are not absolute, meaning that at some point during the year, when, e.g., the demand of regular customers turns out to be lower than expected, the previously reserved amount may be sold to any customer. Third, if a non-regular customer pays a good price, parts of the reserved amounts are usually sold. Therefore, if public forest managers reserve less for regular customers, other consumers are not necessarily able to buy more, but at a lower price.

Another interesting phenomenon is observed when this parameter is set to 100%, i.e., when public forest managers reserve all their sawlogs for regular customers. The sawmill now needs to pay substantially more to obtain sufficient wood. While the increased prices the sawmill pays still do not persuade the domestic public forest managers to provide the sawmill with more wood, the imported amount now increases considerably. This finally leads to an even higher degree of processing capacity utilization than when nothing is reserved for regular customers, but only under the assumption of a high willingness to pay—a situation that probably also would have led to a market exit.

While in section 3.1, the model's extent of replicative and structural validity was analyzed, this section aimed at predictive validity, i.e., showing examples of how the model can be used to predict system behavior that is not yet known (according to the definition of prediction by Kelly et al. [4]).

## 4 Conclusions and outlook

We presented an agent-based model of wood markets in Switzerland, described the validation procedure, and showed to what extent the model is able to reproduce empirical data on amounts, prices, survey results on structural data, and a specific historical market event. The outcome of the rigorous validation qualifies the model to simulate scenarios concerning resource availability and allocation in a given region.

We further showed that ABM is an appropriate modeling method for this type of market, as the system behavior can be modeled as it emerges from the decision behavior of the agents, which is in turn also affected by macro-level variables. The possibility of observing market participants on any level of aggregation is a clear advantage, as we can–for example–check whether not only on average demanders are sufficiently supplied, but also how the supply is distributed on the individual level. Finally, the possibility of modeling transport routes using data from the real road network in the study region is useful, as transportation costs are an important factor for a resource with a relatively low ratio of price per physical mass and volume.

In accordance with Edmonds and Moss [45], we believe that there are two diametrically opposed ways to build a model such as the one presented here: the KISS strategy ("keep it simple, stupid!”) and the KIDS strategy ("keep it descriptive, stupid!"). We decided to use the second approach by creating a complex, but highly descriptive model. This means that we attempted to incorporate as much of our knowledge as possible regarding the market participants and the conditions under which they operate. While this approach makes the model more complex in terms of communication and analysis, it avoids an *a priori* simplification, which may lead to a model that does not include the relevant phenomena [45]. In addition, we experienced that the process of gathering as much data and knowledge as possible during the model-building process can have additional advantages: in our case, the reasons for the failure of the sawmill analyzed in the case study already became apparent before the first simulations were conducted. This shows that not only the model as the final artefact, but also the modeling process, can provide important insights into the system under study, making the journey a considerable part of the reward.

In the future, the model will be used to analyze scenarios relevant to stakeholders and policy makers, concerning–for example–the influence of intermediaries and the effects of set-aside scenarios.

## Supporting information

S1 File Simulation software. The simulation software can be used to replicate the results presented here. All necessary input files are contained inside the file. The results presented in this article are based on the average of 100 runs, using the random seeds 1–100. To run the simulation software with random seed x (where x is an integer number), the following command must be used: "java -Xmx512m -jar S1_File.jar -randomSeed x" (Java must be installed on the system).
(JAR)

S2 File Survey questions. This file contains the relevant survey questions in the original language (German) and English.
(PDF)

S1 Fig Comparison of actual historical and simulated data over time for the assortments considered low validation priority. (TIF)

## References

[1] Bonabeau E. (2002). Agent-based modeling: Methods and techniques for simulating human systems. *Proceedings of the National Academy of Sciences*, 99(suppl 3), 7280–7287. 10.1073/pnas.082080899 PMC128598 12011407

[2] Macal C., & North M. (2014). Introductory tutorial: Agent-based modeling and simulation In *Proceedings of the 2014 Winter Simulation Conference* (pp. 6–20). IEEE Press.

[3] Epstein J. M. (2008). Why model? *Journal of Artificial Societies and Social Simulation*, 11(4), 12.

[4] Kelly R. A., Jakeman A. J., Barreteau O., Borsuk M. E., ElSawah S., Hamilton S. H., et al (2013). Selecting among five common modelling approaches for integrated environmental assessment and management. *Environmental Modelling & Software*, 47, 159–181.

[5] Heath B., Hill R., & Ciarallo F. (2009). A survey of agent-based modeling practices (January 1998 to July 2008). *Journal of Artificial Societies and Social Simulation*, 12(4), 9.

[6] Silver N. (2012). The signal and the noise: *Why so many predictions fail-but some don't* Penguin UK.

[7] Macal C. M. (2016). Everything you need to know about agent-based modelling and simulation. *Journal of Simulation*, 10(2), 144–156.

[8] Janssen M. A., & Ostrom E. (2006). Empirically based, agent-based models. *Ecology and Society*, 11(2), 37.

[9] Schelling T. C. (1971). Dynamic models of segregation. *Journal of mathematical sociology*, 1(2), 143–186.

[10] Axelrod R. (1980). Effective choice in the prisoner's dilemma. *Journal of conflict resolution*, 24(1), 3–25.

[11] Jager W. & Edmonds B. (2015) Policy Making and Modelling in a Complex world In Janssen M., Wimmer M. and Deljoo A. (eds.) *Policy Practice and Digital Science*. Springer, pp. 57–74.

[12] Boero R., & Squazzoni F. (2005). Does empirical embeddedness matter? Methodological issues on agent-based models for analytical social science. *Journal of Artificial Societies and Social Simulation*, 8(4).

[13] van Vliet J., Bregt A. K., Brown D. G., van Delden H., Heckbert S., & Verburg P. H. (2016). A review of current calibration and validation practices in land-change modeling. *Environmental Modelling & Software*, 82, 174–182.

[14] Kostadinov F., Holm S., Steubing B., Thees O. & Lemm R. (2014). Simulation of a Swiss wood fuel and roundwood market: An explorative study in agent-based modeling. *Forest Policy and Economics*, 38: 105–118. doi: 10.1016/j.forpol.2013.08.001

[15] Müller B., Bohn F., Dreßler G., Groeneveld J., Klassert C., Martin, et al (2013). Describing human decisions in agent-based models–ODD + D, an extension of the ODD protocol. *Environmental Modelling & Software*, 48, 37–48. doi: 10.1016/j.envsoft.2013.06.003

[16] Grimm V., Berger U., Bastiansen F., Eliassen S., Ginot V., Giske J., et al (2006). A standard protocol for describing individual-based and agent-based models. *Ecological Modelling*, 198(1–2):115–126. doi: 10.1016/j.ecolmodel.2006.04.023

[17] Grimm V., Berger U., DeAngelis D.L., Polhill J.G., Giske J. & Railsback S.F. (2010). The ODD protocol: A review and first update. *Ecological Modelling*, 221(23):2760–2768. doi: 10.1016/j.ecolmodel.2010.08.019

[18] Holm S., Lemm R., Thees O., & Hilty L.M. (2016). Enhancing Agent-based Models with Discrete Choice Experiments. *Journal of Artificial Societies and Social Simulation*, 19(3), 3 doi: 10.18564/jasss.3121

[19] Laurie A. J., & Jaggi N. K. (2003). Role of 'vision' in neighbourhood racial segregation: a variant of the Schelling segregation model. *Urban Studies*, 40(13), 2687–2704.

[20] Segovia-Juarez J. L., Ganguli S., & Kirschner D. (2004). Identifying control mechanisms of granuloma formation during M. tuberculosis infection using an agent-based model. *Journal of theoretical biology*, 231(3), 357–376. doi: 10.1016/j.jtbi.2004.06.031 15501468 10.1016/j.jtbi.2004.06.031

[21] Evers E., de Vries H., Spruijt B. M., & Sterck E. H. (2011). Better safe than sorry—socio-spatial group structure emerges from individual variation in fleeing, avoidance or velocity in an agent-based model. *PLoS One*, 6(11), e26189 doi: 10.1371/journal.pone.0026189 22125595 10.1371/journal.pone.0026189 PMC3220670

[22] BFS (Swiss Federal Statistical Office) (2015a). *Forstflächen nach Eigentümertyp und Kantonen*, *2014*. Switzerland.

[23] Müller B., Balbi S., Buchmann C. M., De Sousa L., Dressler G., Groeneveld J., et al (2014). Standardised and transparent model descriptions for agent-based models: Current status and prospects. *Environmental Modelling & Software*, 55, 156–163.

[24] McFadden D. (1974). Conditional logit analysis of qualitative choice behavior. *Frontiers in Econometrics*, 105–142.

[25] Sargent, R. G. (2005). Verification and validation of simulation models. Proceedings of the 37th Conference on Winter Simulation, 130–143. Winter Simulation Conference.

[26] Richiardi M., Leombruni R., Saam N., & Sonnessa M. (2006). A Common Protocol for Agent-Based Social Simulation. *Journal of Artificial Societies and Social Simulation*, 9(1).

[27] Windrum P., Fagiolo G., & Moneta A. (2007). Empirical validation of agent-based models: Alternatives and prospects. *Journal of Artificial Societies and Social Simulation*, 10(2), 8.

[28] Page B., Liebert H., Heymann A., Hilty L.M., Häuslein A. (1991): *Diskrete Simulation–Eine Einführung mit Modula-2*. Springer, Berlin, ISBN: 3-540-54421-6

[29] Klügl F. (2008). A validation methodology for agent-based simulations. In *Proceedings of the 2008* *ACM symposium on Applied computing* (pp. 39–43). ACM.

[30] Zeigler B. P., Praehofer H., & Kim T. G. (2000). Theory of modeling and simulation: integrating discrete event and continuous complex dynamic systems Academic press.

[31] Troitzsch K. G. (2004). Validating simulation models In *18th European Simulation Multiconference*. Networked Simulations and Simulation Networks (pp. 265–270).

[32] Railsback S. F., & Grimm V. (2011). *Agent-based and individual-based modeling*: *a practical introduction* Princeton University Press.

[33] Fagiolo, G., Windrum, P., & Moneta, A. (2006). Empirical validation of agent-based models: A critical survey (No. 2006/14). LEM Working Paper Series.

[34] Wiegand T., Jeltsch F., Hanski I., & Grimm V. (2003). Using pattern‐oriented modeling for revealing hidden information: a key for reconciling ecological theory and application. *Oikos*, 100(2), 209–222.

[35] Grimm V., Revilla E., Berger U., Jeltsch F., Mooij W. M., Railsback S. F.,… & DeAngelis D. L. (2005). Pattern-oriented modeling of agent-based complex systems: lessons from ecology. *Science*, 310(5750), 987–991. doi: 10.1126/science.1116681 16284171 10.1126/science.1116681

[36] Scriven M. (1959). Explanation and prediction in evolutionary theory. *Science*, 130: 477–482. 14444298 10.1126/science.130.3374.477

[37] Grünbaum A. (1962). Temporally-asymmetric principles, parity between explanation and prediction, and mechanism versus teleology. *Philosophy of Science*, 29(2), 146–170.

[38] Bianchi C., Cirillo P., Gallegati M., & Vagliasindi P. A. (2007). Validating and calibrating agent-based models: a case study. *Computational Economics*, 30(3), 245–264.

[39] Moss S. (2000). Editorial Introduction: Messy Systems‐The Target for Multi Agent Based Simulation In *Multi-agent-based simulation* (pp. 1–14). Springer Berlin Heidelberg.

[40] WSL / BUWAL (Eidgenössische Forschungsanstalt WSL und Bundesamt für Umwelt, Wald und Landschaft BUWAL) (Eds.) (2001). *Lothar*. *Der Orkan 1999* *Ereignisanalyse*. Birmensdorf, Bern: Eidg. Forschungsanstalt WSL, Bundesamt für Umwelt, Wald und Landschaft BUWAL.

[41] Fehler, M. (2010). Kalibrierung Agenten-basierter Simulationen (Doctoral dissertation, Julius Maximilian University of Würzburg, Germany).

[42] Heckman J. J. (1979). Sample selection bias as a specification error. *Econometrica*, 1979, v47(1), 153–161. doi: 10.2307/1912352

[43] BFS (Swiss Federal Statistical Office) (2015b). Rundholzeinschnitt, Schnitt- und Restholz in den Sägereien in m3, nach Grössenklassen, 2014. Switzerland.

[44] Suedostschweiz (2015). Das Areal der Grosssägerei im Zeitraffer. Retrieved November 26, 2017, from: https://www.suedostschweiz.ch/wirtschaft/2015-10-21/das-areal-der-grosssagerei-im-zeitraffer. Archived at: http://www.webcitation.org/6vGa6c6KH

[45] Edmonds B., & Moss S. (2005) *From KISS to KIDS–An ‘Anti-simplistic’ Modelling Approach* In: Davidsson P., Logan B., Takadama K. (eds) Multi-Agent and Multi-Agent-Based Simulation. MABS 2004. Lecture Notes in Computer Science, vol 3415 Springer, Berlin, Heidelberg.

