# Supplementary material for: Empirical validation of an agent-based model of wood markets in Switzerland
Source: PLoS One. 2018 Jan 19;13(1):e0190605. doi: 10.1371/journal.pone.0190605 (PMC5774711; doi:10.1371/journal.pone.0190605)
Supplement: S2 File — This file contains the relevant survey questions in the original language (German) and English. (PDF) [file pone.0190605.s002.pdf]

Survey Questions

This document lists the relevant survey questions in both English and the original language (German).

Public forest manager survey

Discrete choice experiment

The discrete choice experiment (DCE) conducted in this survey is described in detail in [1].

Question 1

English

What is the average percentage of the annual cut that you reserve for regular customers, even if there is no contract yet?

Sawlogs: _____ Energy wood: _____ Industrial wood: _____

Original Language (German)

Wie gross ist durchschnittlich der Anteil am jährlichen Holzeinschlag (in Prozent), den Sie für bestimmte gute Kunden auch ohne vertragliche Bindung reservieren?

Stammholz: _____ Energieholz: _____ Industrieholz: _____

Question 2

English

Timber harvesting in private forests: percentage of own consumption per assortment (%)?

Sawlogs: _____ Energy wood: _____ Industrial wood: _____

Original Language (German)

Holzernte im Privatwald: Anteil Eigenverbrauch am Sortiment (%)?

Stammholz: _____ Energieholz: _____ Industrieholz: _____

Question 3

English

On average, how many requests to buy wood does your forest enterprise receive annually?

Sawlogs: _____ Energy wood[1]: _____ Industrial wood: _____

[1] Large customers, typically wood chips (i.e. no sale of firewood to small customers)

Original Language (German)

Wie viele Anfragen um Holz zu kaufen gibt es in Ihrem Forstbetrieb durchschnittlich pro Jahr?

Stammholz: _____ Energieholz[1]: _____ Industrieholz: _____

[1] Grosse Kunden, i.d.R. Hackschnitzel (also kein Brennholzverkauf an Kleinkunden)

Question 4

English

On average, how many of these requests are rejected?

Sawlogs: _____ Energy wood[1]: _____ Industrial wood: _____

[1] Large customers, typically wood chips (i.e. no sale of firewood to small customers)

Original Language (German)

Wie viele davon werden durchschnittlich abgelehnt?

Stammholz: _____ Energieholz[1]: _____ Industrieholz: _____

[1] Grosse Kunden, i.d.R. Hackschnitzel (also kein Brennholzverkauf an Kleinkunden)

Sawmill operators survey

Discrete choice experiment

The discrete choice experiment (DCE) conducted in this survey is similar to the DCE with public forest managers described in detail in [1], and differs only in the attributes asked. The attributes and the levels were the following:

English

Attributes	Levels		
Sawlogs in stock[1]	75%	50%	25%
Amount of sawlogs offered (in relation to annual purchase)[2]	5%	1%	0.2%

Original Language (German)

Attribute	Levels		
Lagerfüllstand Rundholz[1]	75%	50%	25%
Menge des angebotenen Rundholzes (Anteil am jährlichen Einkauf)[2]	5%	1%	0.2%

Trust in supplier[3]	0.75 (= rather high)	0.5 (= medium)	0.25 (= rather low)
Buyer's margin[4]	-2 CHF/m^3	+2 CHF/m^3	+6 CHF/m^3

[1] **Sawlogs in stock**. Amount of available sawlogs in stock in relation to the capacity of the stock.

[2] **Amount of sawlogs offered** in relation to annual purchase of sawlogs.

[3] **Trust in supplier.** Trust also includes delivery reliability, the general reputation if the supplier is unknown, etc. There is generally a high degree of trust in regular suppliers (1=very high trust; 0=no trust at all).

[4] **Buyer's margin** for the sawlogs offer currently evaluated. The margin relates to 1 m^3 sawnwood (main product), i.e.:

Margin = Sawnwood revenue – (price of sawlogs + production costs)

Production costs also include transportation costs, salaries, etc. (full cost pricing).

Vertrauen zum Lieferanten[3]	0.75 (= eher hoch)	0.5 (= mittel)	0.25 (= eher gering)
Marge des Holzkäufers[4]	-2 CHF/m^3	+2 CHF/m^3	+6 CHF/m^3

[1] **Lagerfüllstand Rundholz**. Menge des verfügbaren Rundholzes im Lager im Vergleich zur Lagerkapazität.

[2] **Menge des angebotenen Rundholzes** im Vergleich zur gesamten jährlichen Rundholzeinkaufsmenge.

[3] **Vertrauen zum Lieferanten.** Das Vertrauen beinhaltet auch die Lieferzuverlässigkeit, die allgemeine Reputation bei unbekannten Lieferanten etc. Zu Stammlieferanten besteht tendenziell ein hohes Vertrauen (1=Vertrauen sehr gross; 0=gar kein Vertrauen).

[4] **Marge des Holzkäufers** für das aktuell betrachtete Rundholzangebot. Die Marge bezieht sich auf einen Kubikmeter Schnittholz (Hauptprodukt), d.h.:

Marge = Schnittholzerlös – (Rundholzpreis + Produktionskosten)

Produktionskosten enthalten auch Transportkosten, Löhne, etc. (Vollkostenrechnung).

Question 1

English

My maximum stock capacity for sawlogs is _____ m^3

Original Language (German)

Meine Rundholzlagerkapazität beträgt maximal _____ m^3

Question 2

English

On average, the utilized stock capacity in 2014 was _____ m^3

Original Language (German)

Die Auslastung meines Rundholzlagers betrug 2014 im Durchschnitt _____ m^3

Question 3

English

On average, since when do the business relationships to your suppliers exist? Please check, where appropriate!

Duration of business relationship	Public forest manager	Bundling organization	Trader
more than 10 years			
5–10 years			
1–4 years			
less than 1 year			

Original Language (German)

Seit wann bestehen die Geschäftsbeziehungen im Durchschnitt zu Ihren Lieferanten? Bitte ankreuzen!

Dauer der Beziehung	Forstbetrieb	Bündler	Händler
Langjährig (mehr als 10 Jahre)			
Langjährig (5-10 Jahre)			
Mittelfristig (1-4 Jahre)			
Neu (unter 1 Jahr)			

Question 4

English

When buying sawlogs, the percentage of transportation costs on the total costs (per m^3) was in 2014 on average: _____ %

Original Language (German)

Bei der Rundholzbeschaffung betrug der Anteil der Transportkosten an den Gesamtkosten (pro m^3) im Jahr 2014 im Durchschnitt: _____ %

Question 5

English

I buy the vast majority (approx. 90%) of sawlogs from a perimeter of _____ km.

Original Language (German)

Die überwiegende Mehrheit (ca. 90%) meines Rundholzes beziehe ich aus einem Umkreis von _____ km.

Question 6

English

In 2014, from how many suppliers of sawlogs did you receive offers to buy sawlogs?

	1. quarter 2014	2. quarter 2014	3. quarter 2014	4. quarter 2014
Number of Suppliers				

Original Language (German)

Von wie vielen Anbietern von Rundholz erhielten Sie im Jahr 2014 Angebote, um Rundholz zu kaufen?

	1. Quartal 2014	2. Quartal 2014	3. Quartal 2014	4. Quartal 2014
Anzahl Anbieter				

Question 7

English

In 2014, how many suppliers did you ask if they can sell you sawlogs?

	1. quarter 2014	2. quarter 2014	3. quarter 2014	4. quarter 2014
Number of Suppliers				

Original Language (German)

Wie viele Anbieter von Rundholz wurden im Jahr 2014 von Ihnen angefragt, um Rundholz zu kaufen?

	1. Quartal 2014	2. Quartal 2014	3. Quartal 2014	4. Quartal 2014
Anzahl Anbieter				

Question 8

English

Suppliers of sawlogs in 2014:

Suppliers	Number of suppliers in 2014	Total amount supplied (m^3)
Public forest managers		
Bundling organizations		
Traders		

Original Language (German)

Rundholz-Lieferanten im Jahr 2014:

Lieferanten	Anzahl Lieferanten 2014	Gesamte Liefermenge (m^3)
Forstbetriebe		
Bündler		
Händler		

Energy wood buyers survey

Discrete choice experiment

The discrete choice experiment (DCE) conducted in this survey is similar to the DCE with public forest managers described in detail in [1], and differs only in the attributes asked. The attributes and the levels were the following:

English

Attributes	Levels		
Contract duration	5 years	10 years	20 years
Price adjustment mechanism[1]	Yearly adjustment / negotiation	National consumer price index	Woodchips price index of the organization “Holzenergie Schweiz”
Trust in supplier[2]	0.75 (= rather high)	0.5 (= medium)	0.25 (= rather low)
Price of woodchips[3] (1 Rp. = 1/100 CHF)	4.5 Rp/kWh	5.5 Rp/kWh	6.5 Rp/kWh

General remarks concerning the decision situations: The experiment specifically relates to the assortment woodchips. It is assumed that you as a woodchips buyer are concluding a long-term contract with a single supplier (who can have subcontractors, however this is not relevant here).

In the experiment, you have to evaluate several decision situations concerning the conclusion of a contract and decide if you would conclude the contract or not. The delivery quantity and quality of the woodchips are not included as a criterion. However, assume that the contract includes the delivery of the necessary amount and quality in line with the plant operation guidelines.

Original Language (German)

Attribute	Levels		
Laufzeit des Vertrages	5 Jahre	10 Jahre	20 Jahre
Preisanpassungs-mechanismus[1]	Jährliche Anpassung / Verhandlung	Landesindex der Konsumenten-preise	Preisindex Schnitzel von Holzenergie Schweiz
Vertrauen zum Lieferanten[2]	0.75 (= eher hoch)	0.5 (= mittel)	0.25 (= eher gering)
Preis der Hackschnitzel[3]	4.5 Rp/kWh	5.5 Rp/kWh	6.5 Rp/kWh

Vorbemerkung Entscheidungssituationen. Das Experiment beschränkt sich auf das Energieholzsortiment Hackschnitzel. Beim Experiment wird davon ausgegangen, dass Sie als Hackschnitzelkäufer einen langfristigen Vertrag mit einem (einzigen) Lieferanten abschliessen (dieser kann durchaus Unterlieferanten haben; was aber hier keine Rolle spielt).

Beim Experiment geht es darum, dass Sie unterschiedliche Situationen beim Vertragsschluss beurteilen und dann einen Vertrag eingehen oder nicht. Dabei ist die Menge und die Qualität der Hackschnitzel kein Kriterium bzw. Attribut. Gehen Sie davon aus, dass der Vertrag die erfahrungsgemäss jährlich notwendige Menge an Hackschnitzeln in der notwendigen Qualität entsprechend der Richtlinien zum Betrieb der Anlage beinhaltet.

[1] **Price adjustment mechanism.** The national consumer price index measures the price development of a basket of goods and services consumed by private households. It is calculated monthly. The woodchips price index of the organization "Holzenergie Schweiz" integrates price indices of woodchips, mineral oil products, agricultural machines, transportation of goods on the road, and the national consumer price index. It is updated six times a year. Both indices are based on data from the Federal Statistical Office (FSO).

[2] **Trust in supplier.** Trust also includes delivery reliability concerning quality and quantity of woodchips, the general reputation if the supplier is unknown, etc. There is generally a high degree of trust in regular suppliers (1=very high trust; 0=no trust at all).

[3] **Price of woodchips**: The price includes the chipping and the transportation to the storage facility of the customer, the management of the facility, and the disposal of the ash.

[1] **Preisanpassungsmechanismus.** Der Landesindex der Konsumentenpreise misst die Preisentwicklung anhand des sogenannten Warenkorbes, welcher die wichtigsten von den privaten Haushalten konsumierten Waren und Dienstleistungen beinhaltet. Er wird monatlich berechnet. Der Preisindex Schnitzel von Holzenergie Schweiz setzt sich zusammen aus Teilindices für Energieholz-Hackschnitzel, Mineralölprodukte, Landwirtschaftliche Maschinen und Traktoren, Güterverkehr Strasse und dem Landesindex der Konsumentenpreise. Er wird sechs Mal im Jahr aktualisiert. Beide Indices basieren auf Angaben des Bundesamtes für Statistik (BFS).

[2] **Vertrauen zum Lieferanten.** Das Vertrauen beinhaltet auch die Lieferzuverlässigkeit hinsichtlich Qualität und Menge der Hackschnitzel, die allgemeine Reputation bei unbekannten Lieferanten etc. Zu Stammlieferanten besteht tendenziell ein hohes Vertrauen (1=Vertrauen sehr gross; 0=gar kein Vertrauen).

[3] **Preis der Hackschnitzel**: Der Preis beinhaltet das Hacken und den Transport zum Hackschnitzellager beim Verbraucher sowie das Betriebsmanagement der Anlage und die Ascheentsorgung.

Question 1

English

Our current supply contract for woodchips has a duration of (number of years): _____

Original Language (German)

Unser aktueller Hackschnitzel- Liefervertrag hat eine Laufzeit von (Anzahl Jahre): _____

Question 2

English

What were the approximate percentages of softwood and hardwood on the total amount of woodchips in 2014?

Original Language (German)

Wie verteilte sich schätzungsweise die Hackschnitzelmenge 2014 auf Laub- und Nadelholz?

Question 3

English

The maximum woodchips stock capacity of our silo is (loose cubic meters): ______

The maximum woodchips stock capacity of our silo is (number of days at operation under full load): ______

Original Language (German)

Unsere Hackschnitzellagerkapazität im Silo beträgt max. (Srm): ______

Unsere Hackschnitzellagerkapazität im Silo beträgt max. (Anzahl Tage bei Volllastbetrieb): ______

Question 4

English

Since when does the business relationship to your main supplier exist? Please check, where appropriate!

Duration of business relationship	
more than 10 years	
5–10 years	
1–4 years	
less than 1 year	

Original Language (German)

Seit wann besteht die Geschäftsbeziehung im Durchschnitt zu Ihrem (Haupt-) Lieferanten? Zutreffendes bitte ankreuzen!

Dauer der Beziehung	
Langjährig (mehr als 10 Jahre)	
Langjährig (5 -10 Jahre)	
Mittelfristig (1-4 Jahre)	
Neu (unter 1 Jahr)	

Question 5

English

The vast majority (approx. 90%) of woodchips is delivered from a perimeter of (km between forest and plant): ______

Original Language (German)

Den überwiegenden Teil (ca. 90%) unserer Hackschnitzel werden geliefert aus einem Umkreis (Wald – Werk) von (km) : ______

Question 6

English

The percentage of imported woodchips on the total amount purchased was (%): ______

Original Language (German)

Der Importanteil beim Hackschnitzeleinkauf im Jahr 2014 betrug mengenmässig (%): ______

Question 7

English

From how many wood suppliers did you receive an offer of contract to buy woodchips?

Original Language (German)

Von wie vielen Holzanbietern erhielten Sie im Jahr 2014 ein Vertragsangebot, um Hackschnitzel zu liefern?

Question 8

English

How many wood suppliers did you ask in 2014 for a delivery contract?

Original Language (German)

Wie viele Holzanbieter wurden von Ihnen 2014 für einen Liefervertrag angefragt?

References

[1] Holm, S., Lemm, R., Thees, O., & Hilty, L.M. (2016). Enhancing Agent-based Models with Discrete Choice Experiments. *Journal of Artificial Societies and Social Simulation, 19*(3), 3. doi: 10.18564/jasss.3121
