# Supplementary figures and images for: Empirical validation of an agent-based model of wood markets in Switzerland
Source: PLoS One. 2018 Jan 19;13(1):e0190605. doi: 10.1371/journal.pone.0190605 (PMC5774711; doi:10.1371/journal.pone.0190605)

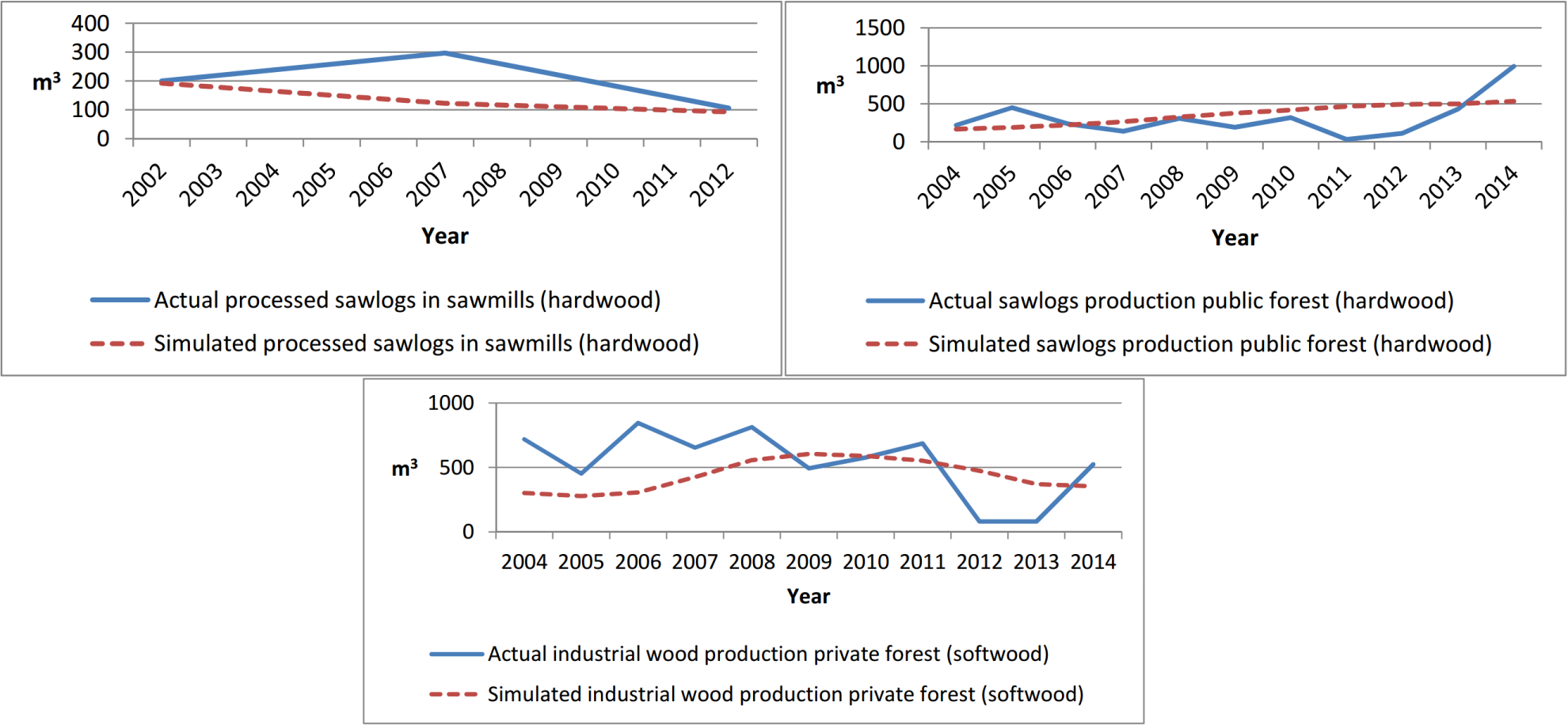

Supplement: S1 Fig — (TIF) [file pone.0190605.s003.tif]
